# Hemicorporectomy in the ICU: a complex case report

**DOI:** 10.1186/s12871-025-03184-x

**Published:** 2025-07-01

**Authors:** Havva Kocayiğit, Burcu Can, Fevzi Sağlam, Ali Fuat Erdem

**Affiliations:** 1 Department of Orthopaedic and Traumatology, Faculty of Medicine, University of Sakarya, Sakarya, Turkey; 2 Department of Anesthesiology and Reanimation, Sakarya University School of Medicine, Sakarya, Turkey

## Abstract

This case report presents the intensive care management of a 65-year-old male patient who underwent hemicorporectomy due to advanced intrapelvic chordoma. Early postoperative complications included supraventricular tachycardia, ventilator-associated pneumonia, and sepsis, while late complications involved wound infection, fluid management challenges, and pleural effusion. Candida parapsilosis was isolated from the wound site, and clinical improvement was achieved with antifungal therapy. After a two-month intensive care stay, the patient was stabilized and transferred to the orthopedic department. This report highlights the importance of a multidisciplinary approach and intensive care management following hemicorporectomy.

**Graphical Abstract:**

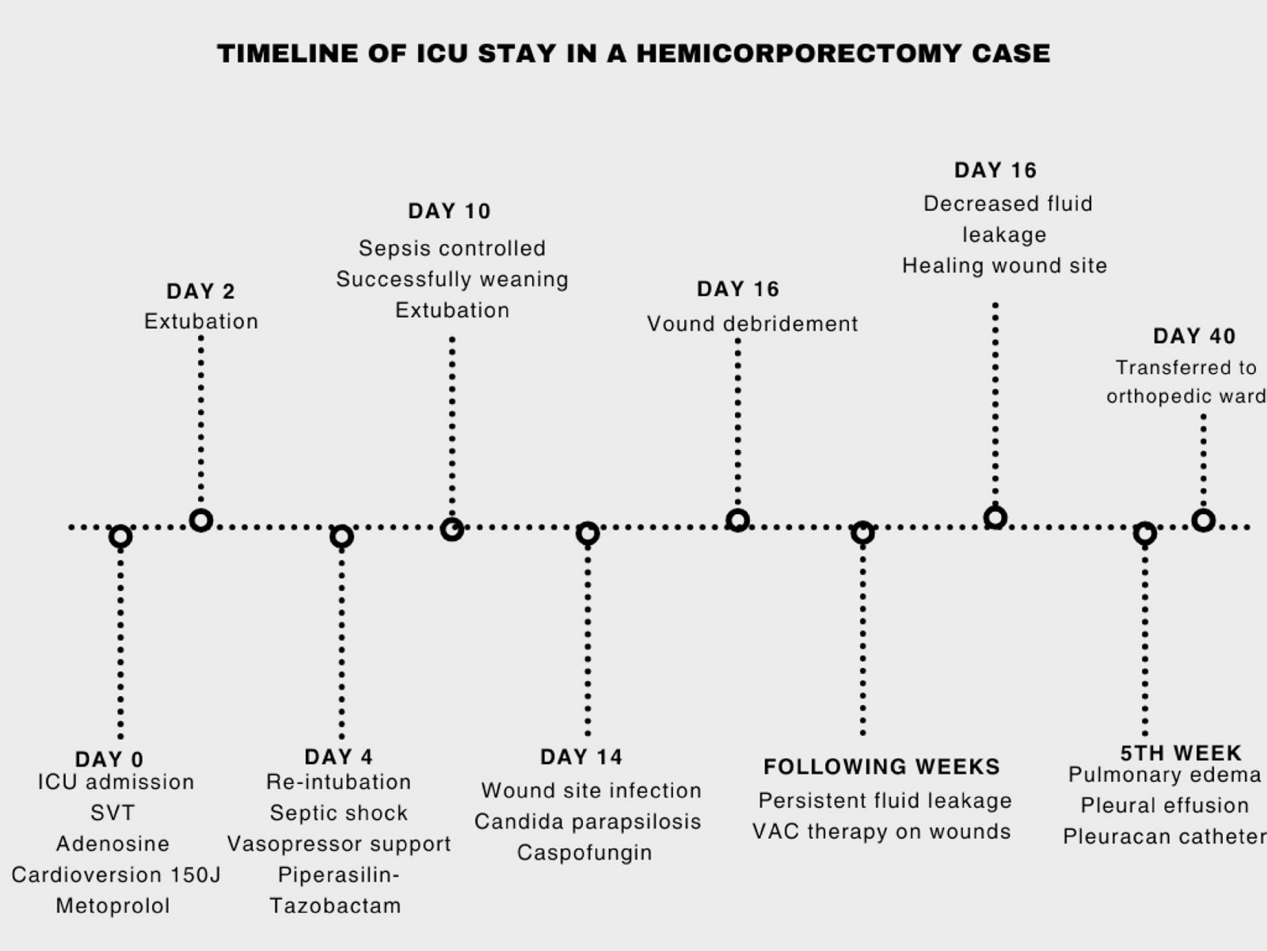

**Supplementary Information:**

The online version contains supplementary material available at 10.1186/s12871-025-03184-x.

## Introduction

Hemicorporectomy is a complex and rare surgery that involves cutting the aorta, inferior vena cava, spinal cord, and involves the removal of the pelvis and lower extremities and creating a nephrostomy for urine and a colostomy for stool. It is typically performed in cases of severe trauma, malignancy, or intractable infections such as osteomyelitis. The procedure is characterized by significant perioperative morbidity and requires careful consideration of patient selection and postoperative care. In the context of intensive care, the management of patients undergoing hemicorporectomy is particularly complex due to extensive physiological changes and the need for multidisciplinary rehabilitation. Postoperative care in the intensive care unit (ICU) is critical for patients who have undergone hemicorporectomy. These patients often present with a range of complications, including hemodynamic instability, infection, and the need for extensive wound care. The procedure necessitates the transection of major vascular structures, including the aorta and inferior vena cava, which can lead to significant blood loss and require meticulous monitoring and management in the ICU setting [1, 2].

Hemicorporectomy has been performed on a very limited number of patients worldwide. Only case reports have been reported in the literature on this subject, and their number is quite limited, and it is very difficult to find descriptive articles on the intensive care process of patients [3, 4]. Our aim in this case report is to share the problems and complications encountered during the intensive care follow-up of a patient who underwent hemicorporectomy.

### Case

**Fig. 1:**
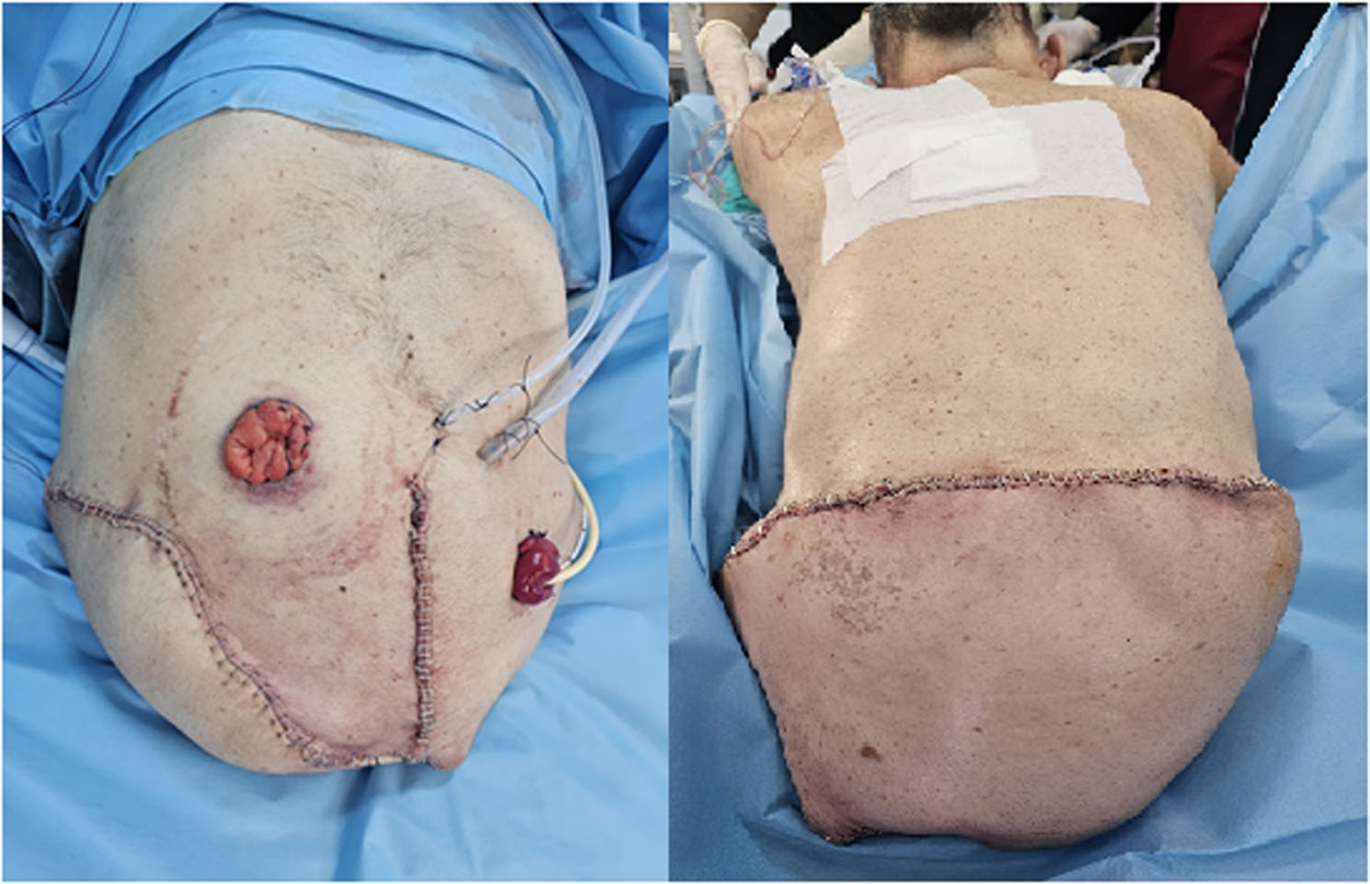
Post-operative view on the operating table

A 65-year-old patient diagnosed with chordoma with intrapelvic spread was admitted to the postoperative anesthesia intensive care unit following a 13-hour hemipelvectomy operation (Fig. 1). The patient was connected to a mechanical ventilator in SIMV mode, and a dexmedetomidine infusion was initiated. Monitoring of EKG, oxygen saturation, and arterial blood pressure was performed (BP: 115/87 mmHg, pulse: 112 bpm, SpO₂: 98%). NICAS was connected for hemodynamic monitoring. The patient was receiving norepinephrine (15 mcg/dk) and dopamine (10 mcg/kg/dk) infusions. In the 3rd hour following the patient’s admission to intensive care, supraventricular tachycardia with a rhythm rate reaching 220 bpm was observed. Adenosine 6 mg was administered with no response and then 12 mg administered but no response again. Due to the patient’s unstable vital signs, cardioverted with 150 J, but again no response was achieved. Following this, 2 mg of metoprolol was given, reducing the patient’s rhythm to 110 bpm. During follow-up, supraventricular tachycardia reoccurred after 6 h and was treated with metoprolol, returning the rhythm to normal. It was considered that the supraventricular tachycardia had developed following inhaled salbutamol treatment, so inhaled salbutamol was not administered again, and no SVT recurred in subsequent monitoring.

On the 2nd day of intensive care stay, the patient was extubated, and high-flow oxygen therapy was initiated. Stool output from the colostomy and urine output from the nephrostomy were monitored. The patient’s vital signs remained stable, but on the 4th day, dyspnea, shortness of breath, rales, increase secretions and fever developed, leading to re-intubation. It was suspected that sepsis developed following pneumonia. CRP and procalcitonin levels began to rise, inotropic and vasopressor support needs increased. The patient, who was on piperacillin-tazobactam, was started on meropenem and vancomycin. Cultures from the tracheal aspirate revealed *Klebsiella* and *Pseudomonas*. During this period, the patient’s platelet count dropped from 220,000 to 30,000. The patient was diagnosed with septic shock and was sedated. The patient in septic shock gradually required less inotropic support, and the levels of CRP and procalcitonin decreased. On the 10th day of hospitalization, the patient, whose septic condition had improved, was weaned off the mechanical ventilator and extubated.

**Fig. 2:**
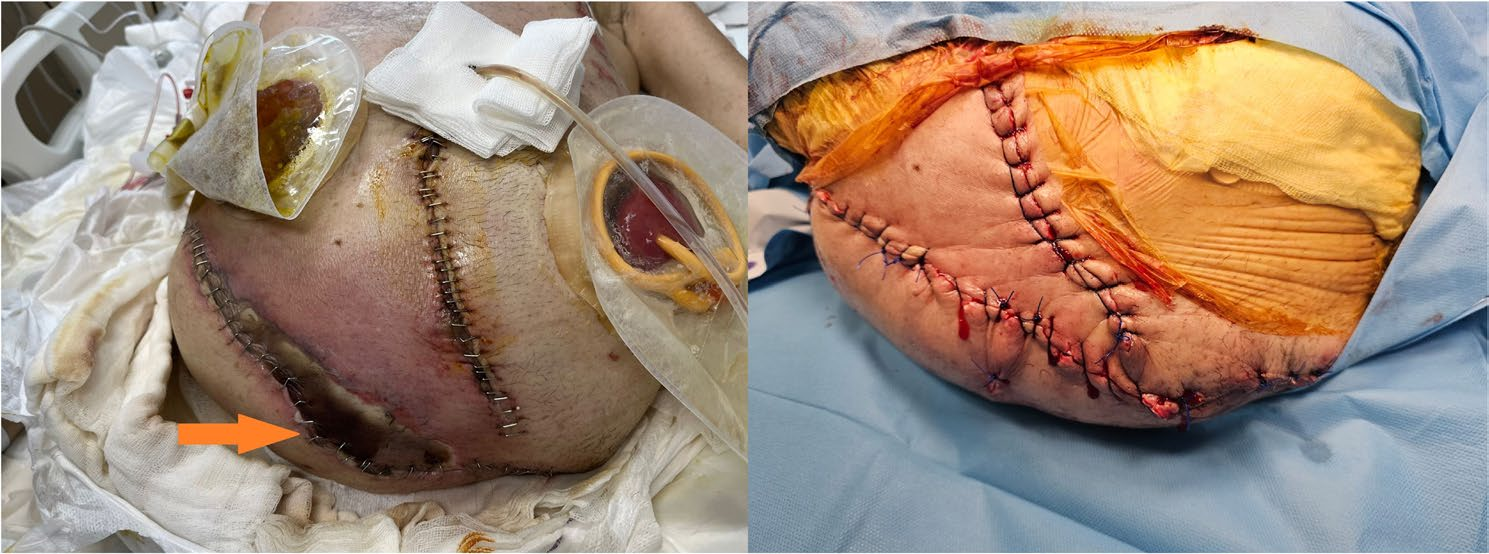
Image of necrotic areas in the wound on the left; image of the wound after debridement on the right

After the patient overcame septic shock, a new episode of fever was observed, along with an increase in procalcitonin and CRP levels. Blood, tracheal aspirate, wound site and urine samples were taken from the patient. There was no growth in the samples, only growth was detected in the wound site. The source of infection this time was the wound site (Fig. 2). Wound dressings were ineffective, and the entire wound site was debrided and cleaned. Microbiology reported that there was a fungal signal from the wound sample and empirical caspofungin treatment was initiated. After 2 days, Candida *parapsilosis* was identified and that it was sensitive to fluconazole, caspofungin and micafungin. The antifungal was not changed. After treatment with caspofungin and wound debridement, the wound infection healed. Wound healing took a prolonged time due to two reasons. The first was the development of necrotic areas at the infection site and small necrotic parts on the flap. The second reason was the discharge of third-space fluid from the wound site.

**Fig. 3:**
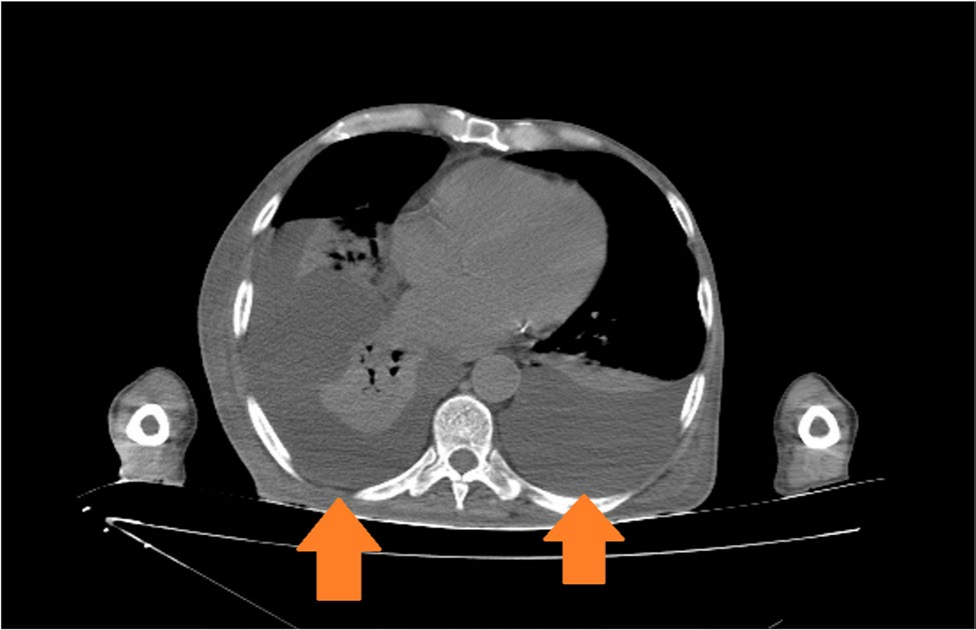
Pleural effusion image on thorax CT

**Fig. 4:**
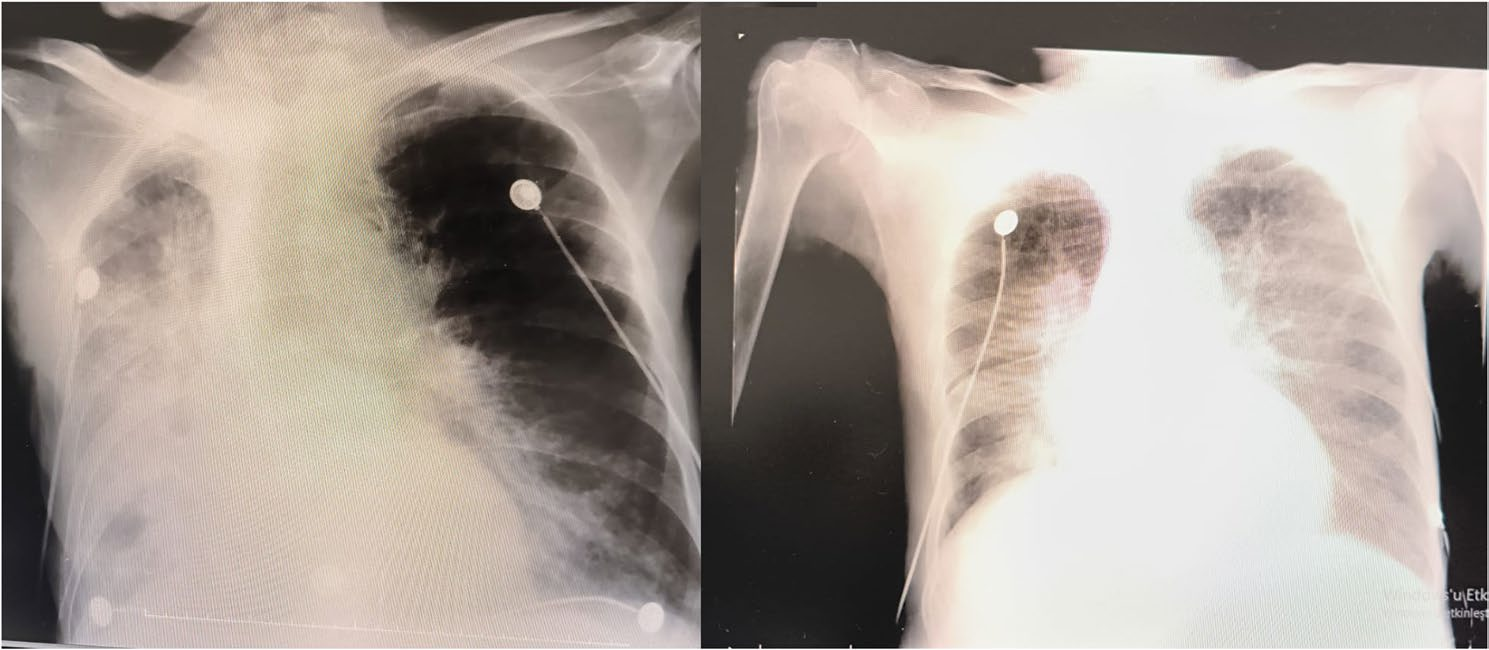
On the left, pleural effusion on the pa chest radiograph; on the right, pa chest radiograph after evacuating thoracentesis

One of the most significant issues encountered during the patient’s ICU stay was fluid management. When fluid restriction was applied, the patient experienced significant fluid loss, leading to a drop in blood pressure and the need for inotropic support. On the other hand, when fluid restriction was not implemented, there was an increase in free fluid in the abdominal cavity, which drained from the wound sites, also the patient developed pulmonary congestion, resulting in complaints of dyspnea (Figs. 3 and 4). It was difficult to maintain the patient’s fluid replacement therapy and stabilise his hemodynamics. During this period, PICCO could not be applied to monitor fluid therapy because there was no femoral vein to insert CVP. Similarly, NICAS did not provide accurate information because it did not have a large part of the body. Therefore, daily ultrasound and lung and vena cava diameter measurements were used in the patient’s follow-ups. In this respect, ultrasound was very useful in monitoring both pulmonary edema and pleural effusion fluid and in evaluating the patient’s overall volume load.

The constantly moist skin tissue due to the continuous serious fluid drainage from the wound site was problematic. As a result, VAC therapy was initiated on the wounds, and the goal was to achieve gradual closure of the wounds in sections. As the hospitalization progressed, the fluid leakage into the third space decreased, and by the end of the second month, most of the wound sites had closed. Only a 3 cm area on the patient’s back, corresponding to a pressure ulcer with poor circulation, showed delayed closure.

However, the patient continued to experience intermittent pulmonary edema and pleural effusion. Every two weeks or so, therapeutic thoracentesis or pleurocaine was performed due to significant pleural effusion. The patient’s echocardiogram revealed an ejection fraction of 45%. It was assessed as mild heart failure; however, it was noted that it would not pose a significant problem given the patient’s weight.

**Fig. 5:**
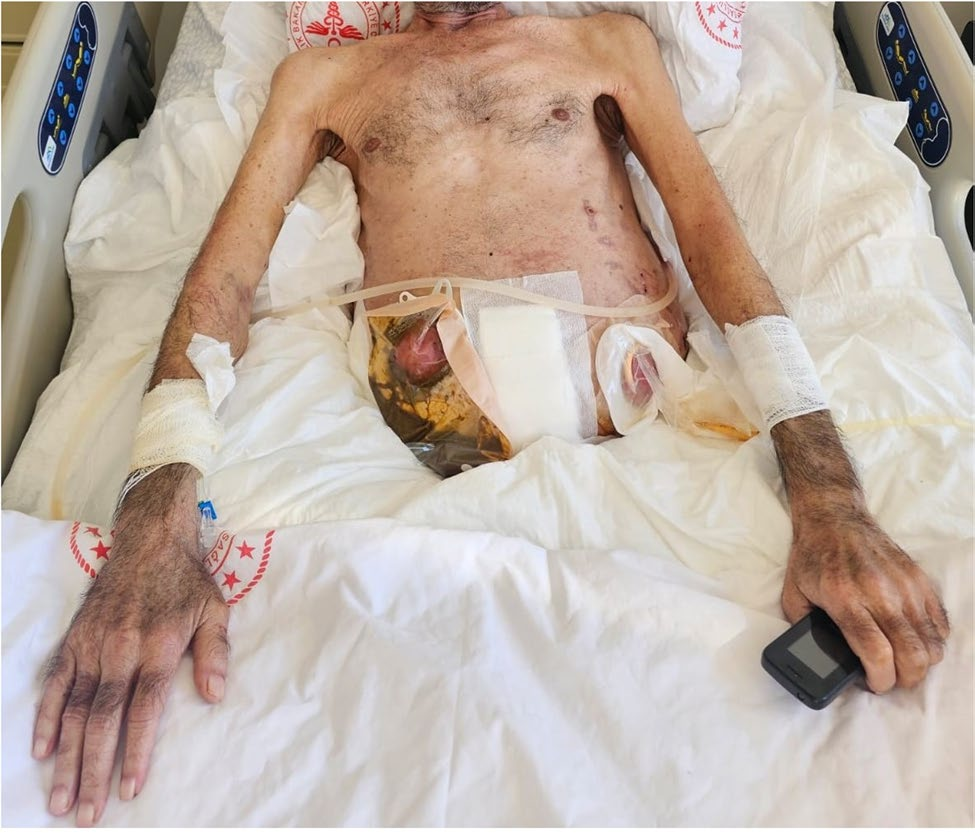
Image of the patient going to the orthopedic ward

After being monitored in the intensive care unit for 40 days, the patient is oriented, cooperative, and stable with respect to vital signs, and was transferred to the orthopedic department (Fig. 5).

## Discussion

Hemicorporectomy is a complex surgical procedure that offers a solution for severe medical conditions; however, it requires careful evaluation of hemodynamic changes and postoperative complications. This surgery can complicate patient monitoring in the intensive care unit (ICU) and often necessitates prolonged follow-up. In our case, early postoperative challenges included arrhythmia and difficulty with weaning, while later complications involved sepsis, fluid management issues, and wound infection.

Shortly after the patient was admitted to the ICU, we encountered an arrhythmia that required cardioversion. We observed that this condition developed following the administration of salbutamol, which was subsequently discontinued. Salbutamol, a selective β2-adrenergic agonist, is primarily used as a bronchodilator in the management of asthma and chronic obstructive pulmonary disease. Although relatively selective for β2-adrenergic receptors, it can also stimulate β1-adrenergic receptors in the myocardium, particularly at higher doses. This stimulation may increase myocardial contractility and heart rate, potentially elevating myocardial oxygen demand and causing arrhythmogenic effects such as tachycardia and atrial fibrillation [5]. A study by Jasiński et al. demonstrated that nebulized salbutamol could alter atrial electrical properties in critically ill patients, suggesting that even therapeutic doses may significantly affect cardiac rhythm [6]. Following the hemicorporectomy, the patient’s weight decreased from 75 kg to 40 kg, raising the possibility that the salbutamol dose administered postoperatively was excessive relative to the new body mass.

Wound infections following amputation are a significant clinical concern. Reported infection rates vary widely, ranging from 13 to 57% depending on the patient population and clinical context [7]. In a study by Das et al. investigating postoperative wound infections in orthopedic surgery, the most commonly isolated pathogen was Staphylococcus aureus. However, fungal pathogens were also identified, with Aspergillus niger found in 2.5% of cases and Candida albicans in 5.5% [8].

In our patient, we believe bacterial growth at the wound site was suppressed due to ongoing antibiotic therapy for ventilator-associated pneumonia and sepsis. Nonetheless, considering the patient’s compromised immune status, the development of small necrotic areas at the wound-flap junction, the prolonged surgical duration, and the possibility of compromised sterility during the procedure, we suspect the wound became colonized by fungal organisms. Wound dressings failed to improve the clinical condition, but rapid healing was observed following surgical debridement. In a previously reported case of hemicorporectomy, Candida albicans was similarly isolated from the surgical site, and the patient responded well to antifungal therapy [9].

Hemicorporectomy, which involves the removal of the pelvis and lower extremities, significantly alters the patient’s hemodynamic profile, potentially leading to instability and changes in blood flow dynamics. Postoperatively, patients may experience changes in cardiac output and systemic vascular resistance due to the loss of lower body mass and alterations in venous return. The removal of the lower extremities reduces venous capacitance, which can affect preload and, consequently, cardiac output [9]. In our case, we initially anticipated hypertension and pulmonary edema due to decreased venous capacitance. However, during the first two weeks, the patient remained hypotensive due to sepsis and surgical site infection, at times requiring norepinephrine support. After the resolution of the infectious process, the patient developed hypertension, pulmonary edema, and pleural effusion. A pleural catheter (pleuracan) was inserted, followed by several therapeutic thoracenteses. Despite these interventions, the issue has not been fully resolved; the patient still occasionally experiences dyspnea and pulmonary edema (at the 7th postoperative month), which improves with diuretic therapy. 

In conclusion, hemicorporectomy is an extremely complex and demanding procedure, both surgically and from an intensive care perspective. A multidisciplinary approach during the ICU stay, just as in the operating room, is essential for optimizing outcomes.

## Supplementary Information

Supplementary Material 1

Supplementary Material 2

## Data Availability

No datasets were generated or analysed during the current study.
