# Supplementary material for: Hemicorporectomy in the ICU: a complex case report
Source: BMC Anesthesiol. 2025 Jul 1;25:304. doi: 10.1186/s12871-025-03184-x (PMC12210823; doi:10.1186/s12871-025-03184-x)
Supplement: Supplementary file 1 — Supplementary Material 1 [file 12871_2025_3184_MOESM1_ESM.docx]

**Full title: Hemicorporectomy in the ICU: A Complex Case Report**

**Short Title: Hemicorporectomy in the ICU**

Havva KOCAYİĞİT^1^, Burcu CAN^1^, Fevzi SAĞLAM^2^, Ali Fuat ERDEM^1^

1 Department of Anesthesiology and Reanimation, Sakarya Education and Research Hospital, Sakarya, Turkey

2 Department of Orthopaedic and Traumatology, Faculty of Medicine, University of Sakarya, Sakarya, Turkey

**Address for correspondence:**

Havva Kocayigit, MD

Department of Anesthesiology and Reanimation, Sakarya University School of Medicine

Sakarya, Turkey

Tel: +90 542 595 00 20

e-mail: [havvakocayigit@gmail.com](mailto:havvakocayigit@gmail.com)

**Statement of authorship:** All authors take responsibility for all aspects of the reliability and freedom from bias of the data presented and their discussed interpretation

**Funding:** none

**Conflict of interest:** none

**Grammar editing done by scribendi.**
