# Supplementary figures and images for: Hemicorporectomy in the ICU: a complex case report
Source: BMC Anesthesiol. 2025 Jul 1;25:304. doi: 10.1186/s12871-025-03184-x (PMC12210823; doi:10.1186/s12871-025-03184-x)

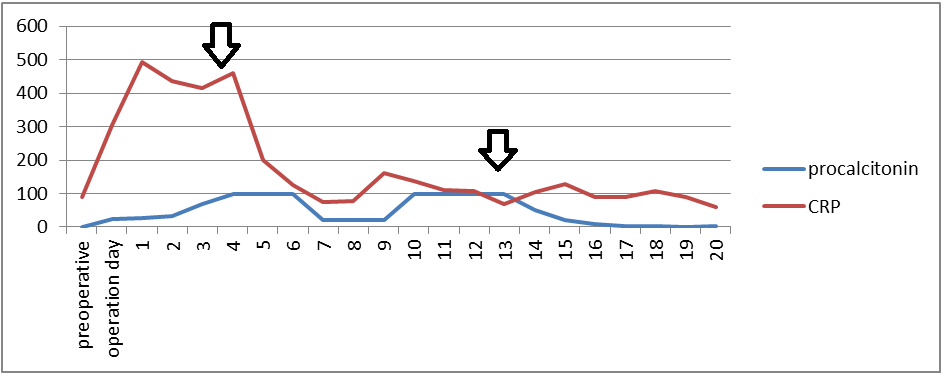

Supplement: Supplementary file 2 — Supplementary Material 2 [file 12871_2025_3184_MOESM2_ESM.png]
